# Characterisation of Asphalt Concrete Using Nanoindentation

**DOI:** 10.3390/ma10070823

**Published:** 2017-07-18

**Authors:** Salim Barbhuiya, Benjamin Caracciolo

**Affiliations:** Department of Civil Engineering, Curtin University, Perth 6845, Australia; benjamin.caracciolo@graduate.curtin.edu.au

**Keywords:** asphalt concrete, hydrated lime, nanoindentation, Young’s modulus, hardness

## Abstract

In this study, nanoindentation was conducted to extract the load-displacement behaviour and the nanomechanical properties of asphalt concrete across the mastic, matrix, and aggregate phases. Further, the performance of hydrated lime as an additive was assessed across the three phases. The hydrated lime containing samples have greater resistance to deformation in the mastic and matrix phases, in particular, the mastic. There is strong evidence suggesting that hydrated lime has the most potent effect on the mastic phase, with significant increase in hardness and stiffness.

## 1. Introduction

Asphalt concrete (AC) is widely used around the world due to its primary function in road and pavement construction. AC is a composite material composed of a heterogeneous mix of asphalt binder and mineral aggregates. It is generally agreed that AC consists of three distinct phases: mastic, matrix, and aggregate phases [1]. The mastic phase is defined by asphalt binder interacting with fines (particles passing the 0.075 mm sieve) to form a thin film that coats and binds the aggregate particles. The matrix phase is defined as the mix of asphalt binder and fillers passing the 4.75 mm sieve. 

The capacity to study the mechanical behaviour of materials is expanding with the introduction of new technologies that can probe locally at nanoscale resolutions and with great control over experimental variables. Using nanoindentation, a multi-structured, multi-phased material like AC can be examined under a variety of loading conditions. Whilst nanoindentation has been used as a means of testing material properties for decades [2,3,4], studies exploring its use on asphalt binder and AC have until recently been limited [5,6,7]. There are difficulties in performing the test on asphalt binder due to the soft and highly viscoelastic nature of the material [8]. It was found that the use of a spherical indenter was better suited to the binder, but the Berkovich indenter tip was able to produce successful results on concrete [9]. 

In order to modify the properties of asphalt binder and asphalt concrete mixes various fillers (aggregate particles fine than 75 μm) are added. Some of the commonly used fillers include Portland cement, hydrated lime, fly ash, limestone dust and clay particles [10]. It is well documented that filler exerts a significant effect on the characteristics and performance of AC mixes [11,12]. It is believed that good packing of coarse, fine, and filler aggregates provides a strong backbone for the AC mix [13]. The presence of filler in AC mixes is even more important because of its possible interaction with asphalt. Owing to the larger surface area, fillers absorb more asphalt and its interaction with asphalt leads to better performance of AC mixes. Fillers are also found to improve the temperature susceptibility and durability of asphalt binder and AC mixes [14].

Calcium hydroxide, known more commonly as hydrated lime, is one of the fillers frequently used in AC to improve the asphalt-aggregate bond and the binder’s resistance to water-induced damage [15,16,17,18,19]. However, limited research is available on its effects on load-displacement behaviour and nanomechanical properties of individual AC phases. In this study, nanoindentation is used to assess the load-displacement characteristics of AC with and without hydrated lime and obtain the nanomechanical properties in terms of Young’s modulus and hardness in the mastic, matrix, and aggregate phases. 

## 2. Experimental Methods

### 2.1. Materials 

Two hot mix AC were selected for testing in this study (one with hydrated lime and one without). Each mix contained a heterogeneous blend of crushed granite aggregates (10 mm) and asphalt binder. A 10 mm size was chosen as this is a common fine aggregate size used on roads, pathways and car parks. The aggregates were sourced from Boral and BGC and dried in the Geomechanics Lab of Curtin University Australia. The standard addition of hydrated lime in AC mixes in Australia is 1.5% of the total weight of the dry mix [20]. Therefore, in this study the hydrated lime was added at 1.5%. The mix details are summarised in Table 1. The term “base” in Table 1 refers to a mix not containing hydrated lime. Other properties of the mixes are given in Table 2. 

### 2.2. Sample Preparation

The samples were mixed and compacted as per AS 2150–2005 [21]. The compacted cylindrical samples were then cut down to smaller size in order for them to fit the maximum thickness requirements of the indenter. The surfaces of the samples were sliced off using a concrete cutter and then divided into 10 mm cubic sections using a precision saw for the recommended nanoindentation size. Four samples from each mix were selected for resin casting, a process that suspends the sample in resin, creating total stability for grinding, polishing, and nanoindentation. Once the resin solidified 24 h later, the samples were ground using a grinder-polisher machine. Before polishing, a step called impregnation was used. The process of impregnation helped to fill the valleys in the structure of the sample, creating an even surface. The samples were covered with a thin coat of EpoThin and placed in a vacuum chamber, eradicating all air bubbles. Following this, the samples were grinded once again in order to expose the surface. Preparation of the samples is complete after the final stage of polishing. Four decreasing sizes of Polycrystalline Diamond Suspension (9 µm, 6 µm, 3 µm, and 1 µm) were used to lubricate a polishing cloth, which rotates under the samples. The diamond particles consist of single grains that have sharp edges, working to remove excess particles and enabling a smooth surface finish.

### 2.3. Nanoindentation

**Figure 1:**
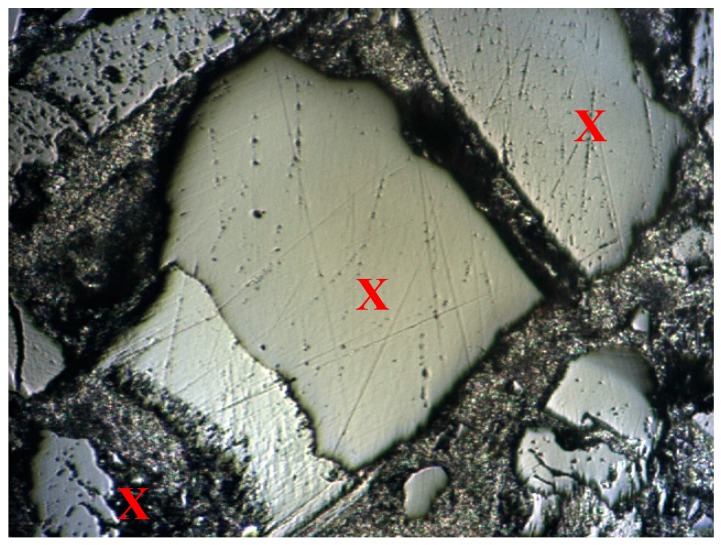
Indentation sites for Set 1 (marked with X).

Nanoindentation tests were carried out using Agilent Nano Indenter G200 (Keysight Technologies, Inc., Santa Rosa, CA, USA). Poisson’s ratio for both AC and indenter tip were assumed based on average values from previous literature [22]. In order to appropriately represent the diversities that exist within AC, it was important to perform a sufficient number of indents over a variety of areas for each sample. The following five sets of tests were conducted using nanoindentation:

Set 1: Two indents on a randomly selected area of mastic and two indents on a randomly selected area of aggregate (Figure 1). This was the initial test to assess the loading conditions and whether the 20 s dwell time was sufficient to minimise the effects of creep. It provides a basic reference for the mastic/aggregate characteristics of each sample, although four indents are not representative of the overall sample.

**Figure 2:**
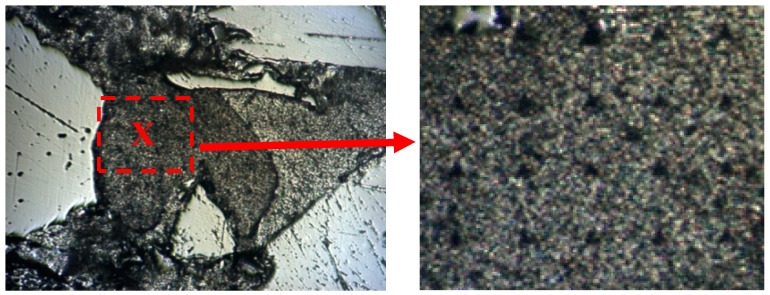
Indentation sites for Set 2 (marked with X).

Set 2: A 10 × 10 grid (100 indents) on a manually selected area of each sample containing mastic, matrix, and aggregate (Figure 2). The grid was organised with each indent spaced at 40 µm apart in order to avoid residual impressions from previous indents. Areas were chosen such that there was a relatively even mix of each phase present in order to gather a good representation of data. 

## 3. Results and Discussion

### 3.1. Load-Displacement Characteristics

**Figure 3:**
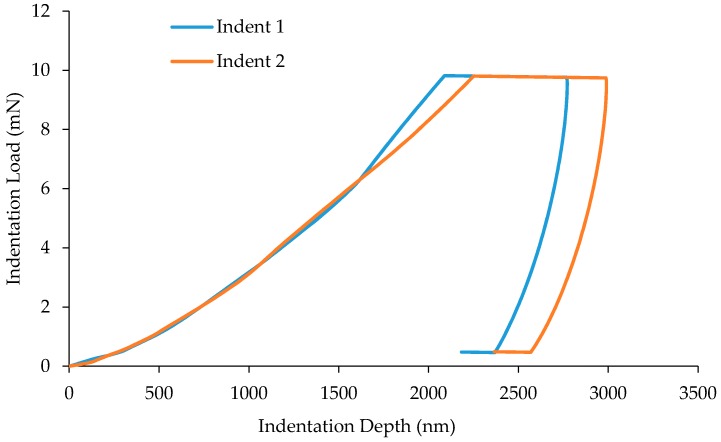
Load-displacement curve for two separate indents on mastic phase.

Figure 3 shows the load-displacement characteristics of two separate indents on the mastic region. First, it is evident that both curves in Figure 3 have an almost identical shape, with one displacing more during loading. It is also evident that there is a period of creep during maximum load, which was expected given the viscoelastic nature of the mastic. However, the unloading curve appears to elicit an elastic response, which indicates the dwell period of 20 s was enough to offset the effects of the creep. An elastic unloading curve is necessary in order to use the Oliver-Pharr method [23] of analysis. Both curves are set to 95% unloading. This means when the indenter unloads to approximately 0.5 mN, a further dwell period of 75 s is employed in order to account for thermal drift. The horizontal line at the end of the unloading curve is a measure of the displacement during this period and accounted for automatically as drift correction. This is the case for all indentations performed. Figure 3 also shows a difference of approximately 200 nm in indentation depth between the two tests. The diversity within the mastic means it is highly unlikely that two indentation points will be exactly the same, even within the same region.

**Figure 4:**
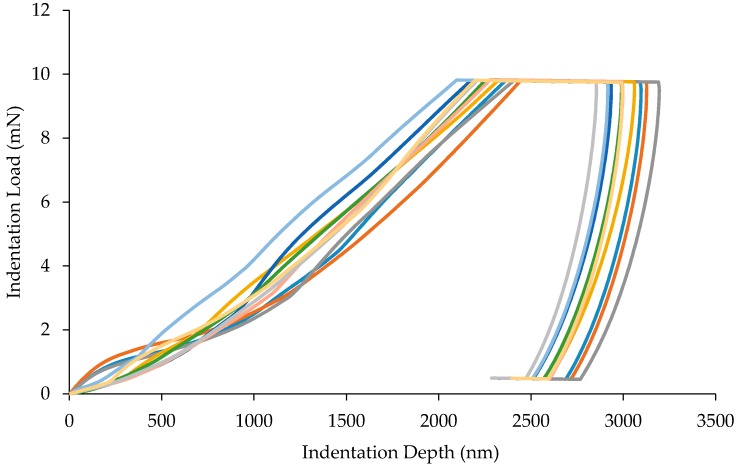
Load-displacement curve for 10 indents on mastic phase.

**Figure 5:**
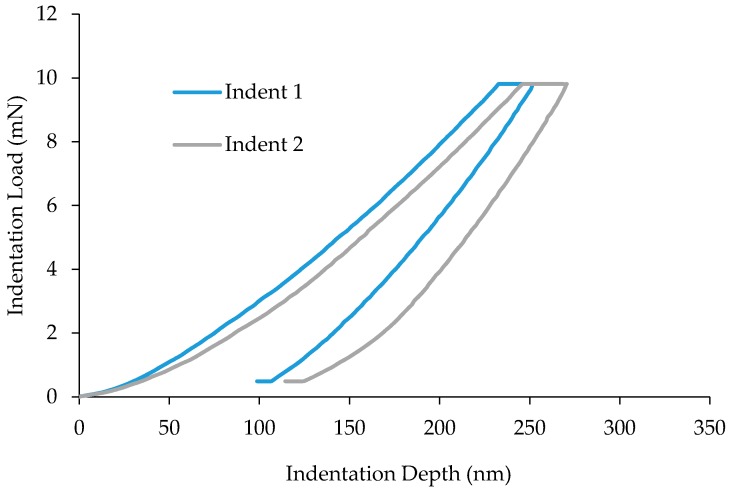
Load-displacement curve for two separate indents on aggregate phase.

**Figure 6:**
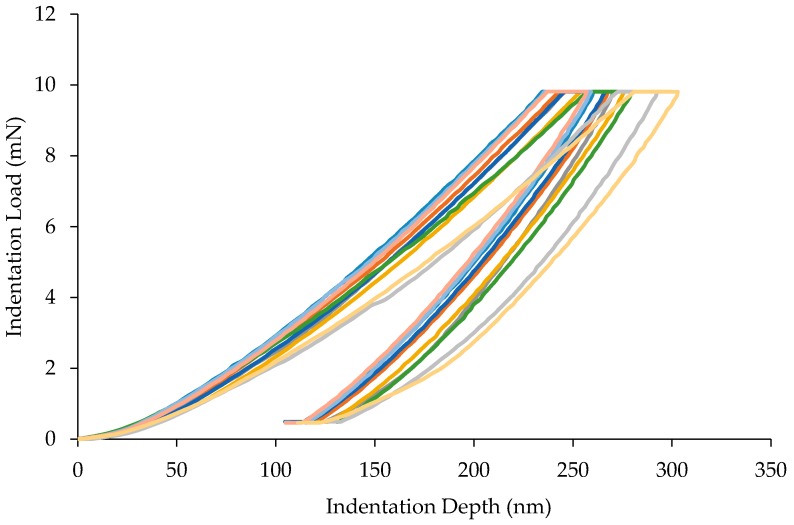
Load-displacement curve for 10 indents on aggregate phase.

Figure 4 illustrates the diversity within the mastic by showing 10 indents. It is notable that the shape of the unloading curves for the tests in Figure 5 and Figure 6 are consistent in having an elastic response. There is a noticeable amount of hysteresis at the beginning of most curves before 1000 nm depth. After this, the data normalises and follows a reliable shape. It is possible that this is due to imperfections on the surface before the indenter reaches material with relative consistency.

Figure 5 shows two separate indents performed on the aggregate phase of the sample. The characteristics of the aggregate curves compared to those on the mastic are evident in the reduction of indentation depth and lower creep response during the dwell period. Indentation depth at the peak load of 10 mN is in the range of 200–300 nm indicating a much harder material when contrasted with the 2000–3000 nm range of the mastic. Figure 6 displays 10 tests performed on a section of aggregate, spaced 40 µm apart. The overall variance between indents on a particular region of aggregate is also less apparent as can be seen in Figure 6. Over the 10 tests, the change in indentation depth at max load was just 72 nm, which is indicative of a fairly consistent aggregate material. This can also be seen in the “smoothness” of the loading curve compared to those on the mastic.

**Figure 7:**
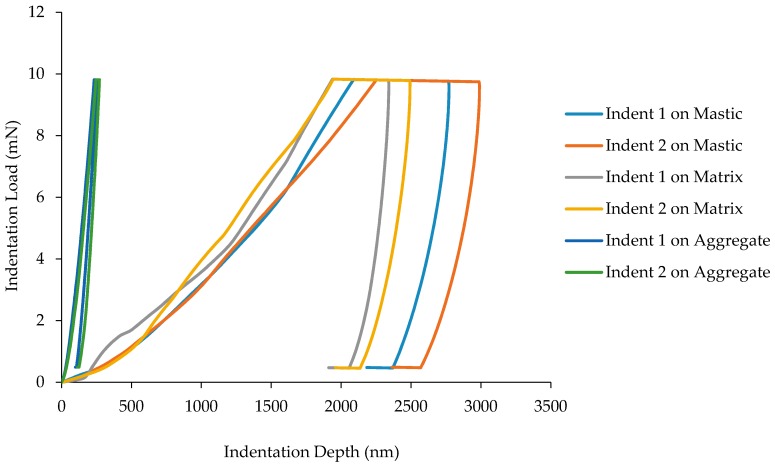
Load-displacement curve for all phases in AC without hydrated lime.

The relative difference of each of the phases in AC without hydrated lime is more apparent when displayed on the same scale as shown in Figure 7. There is a clear distinction between the aggregate and mastic/matrix phases when looking at the indentation depth and creep during dwell time. The loading curves for both the mastic and matrix phases show a much greater elastic-plastic response than the aggregate due to the softness of the regions. It can also be seen that the displacement during dwell time for the mastic phase lessens during the matrix phase and is relatively small during the aggregate phase. The unloading portion of the load-displacement curves for each region displays elastic flow.

**Figure 8:**
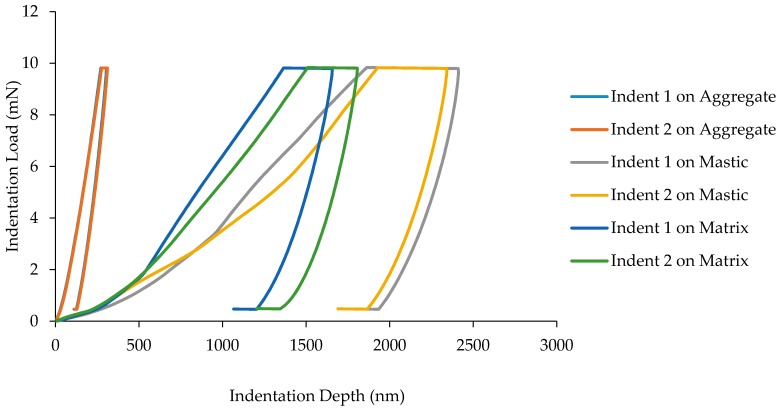
Load-displacement curve for all phases in AC with hydrated lime.

Figure 8 shows a series of indentations taken on the sample containing hydrated lime. It appears that the addition of hydrated lime to the mix does not directly affect the elastic-plastic properties of the loading curve or the elastic response of the unloading curve. What is immediately apparent, however, is the reduction in displacement compared to the sample without the lime. Considering the documented effects of hydrated lime as a stiffening agent, this is an expected result. The hydrated lime addition appears to have a greater effect on displacement in the mastic and matrix phases. This is logical considering the additive bonds itself with asphalt binder, which is mostly present within these phases. 

**Figure 9:**
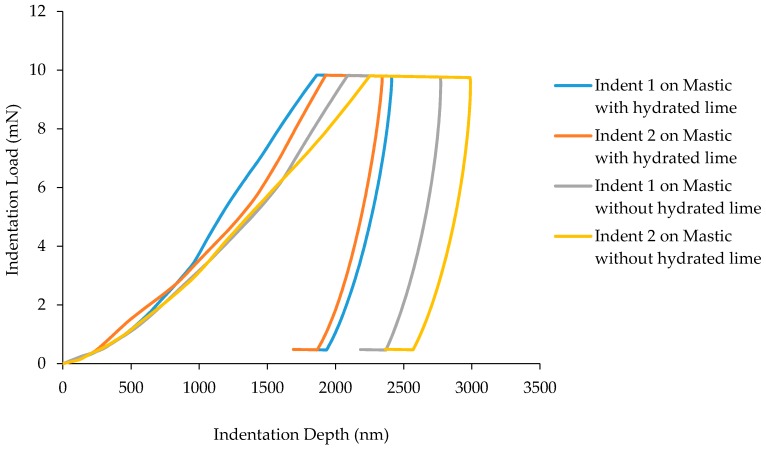
Load-displacement curve for mastic phases with hydrated lime.

**Figure 10:**
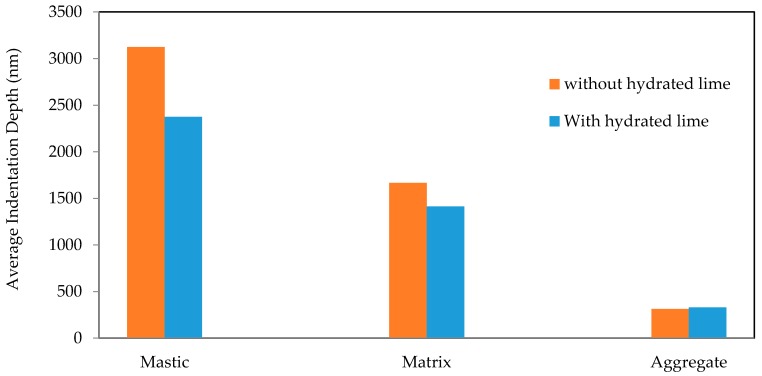
Average indentation depth for all phases.

Figure 9 displays nanoindentation tests performed on the mastic phase of samples with hydrated lime. It can be seen from the figure that the difference in indentation depth between samples with and without hydrated lime is obvious. However, comparing a small number of indents does not necessarily provide an accurate representation of a material with the complexities of asphalt concrete. Figure 10 presents the results of two 10 × 10 grids of indentations for both the samples. The result is an average of 186 successful indents for the hydrated lime containing sample and 167 successful indents for the sample without hydrated lime. 

Overall, there is reasonable confirmation that the hydrated lime containing samples have greater resistance to deformation in the mastic and matrix phases, in particular, the mastic. Again, there appears to be a negative differential in the aggregate phase, although 16 nm could be accounted for as natural variance within the material. There is certainly no indication from the results that hydrated lime acts as a stiffener in the aggregate phase.

### 3.2. Nanomechanical Properties

**Figure 11:**
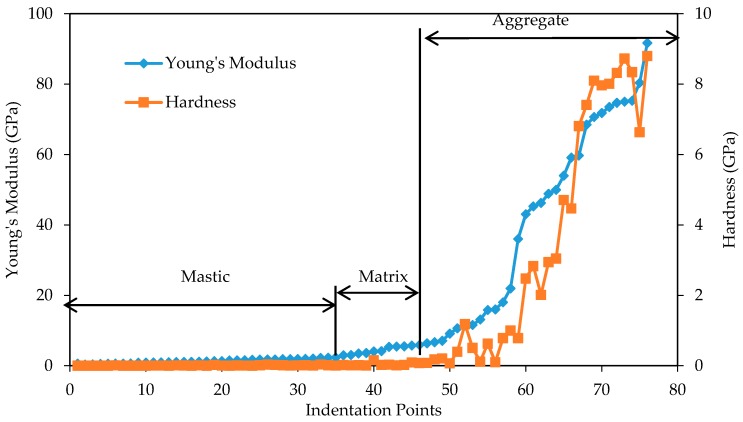
Young’s modulus and hardness of all phases in sample without hydrated lime.

**Figure 12:**
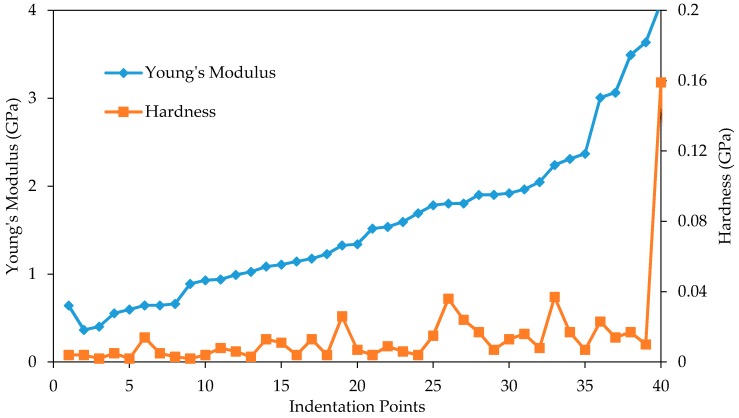
Young’s modulus and hardness in mastic phase of sample without hydrated lime.

The nanomechanical properties (Young’s modulus and hardness) in sample without hydrated lime are shown in Figure 11. It can be seen that there is a trend of increasing hardness as Young’s modulus increases. The Young’s modulus range seems to be under 4 GPa for mastic, 4–12 GPa for matrix, and 12–100 GPa for aggregate. The aggregate phase in particular has a wide range of Young’s modulus and hardness values compared to the other phases. Figure 12 takes a closer a look at the Young’s modulus and hardness values of the mastic region. The hardness still appears to increase slightly with the Young’s modulus, although it is less clear at this scale.

**Figure 13:**
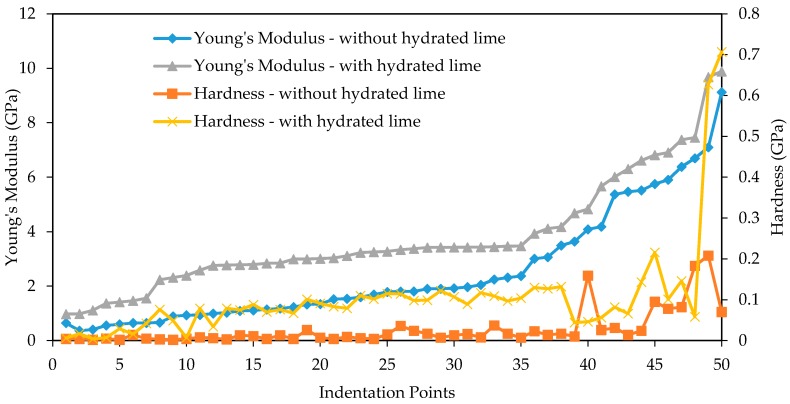
Young’s modulus and hardness on mastic and matrix phase for samples with and without hydrated lime.

Figure 13 compares the Young’s modulus and hardness on mastic and matrix phase for samples with and without hydrated lime. There is a clear observable trend for the sample containing hydrated lime having higher Young’s modulus throughout both phases. Similarly, the majority of hardness values are higher for the hydrated lime containing sample, with the exception of point 40 which appears to be an outlier, and point 48. The difference between the samples decreases from point 35, which is close to when the indents begin appearing in the matrix phase. There is some indication that the hydrated lime has less of an effect on Young’s modulus and hardness in the matrix region. It should be clarified that since this data was processed from the 10 × 10 grid of indentations, the number of indents for mastic/matrix/aggregate was not the same for both samples. In fact, the mastic region tested on the sample containing hydrated was larger and had a greater quantity of indents. In order to directly compare the different phases, some of the mastic data for samples with hydrated lime was omitted. The omitted indents display similar hardness/modulus characteristics to those included and do not contradict observations from Figure 13.

**Figure 14:**
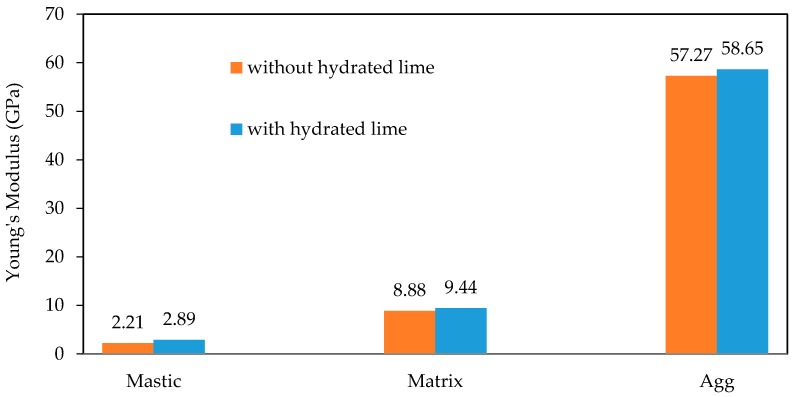
Average Young’s modulus values obtained from the indentation grids.

**Figure 15:**
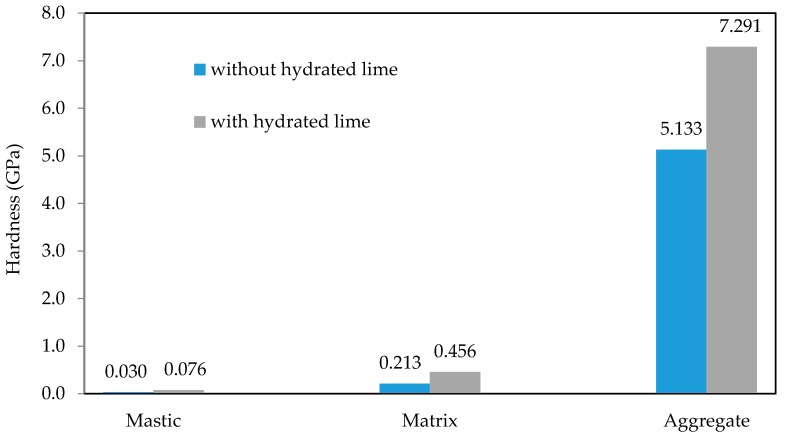
Average hardness values obtained from the indentation grids.

Figure 14 and Figure 15 shows the average Young’s modulus and hardness values respectively obtained from the indentation grids. The Young’s modulus and hardness for samples with hydrated lime are higher than those of the samples without them. In mastic phase there is an increase of 31% in the Young’s modulus and 153% in the hardness due to the addition of hydrated lime. In matrix phase there is an increase of 6% in the Young’s modulus and 114% in the hardness due to the addition of hydrated lime.

## 4. Conclusions

Using nanoindentation with a Berkovich indenter, it was possible to successfully capture the heterogeneity of asphalt concrete, with distinctly different properties for mastic, matrix, and aggregate phases. A dwell time of 20 s was sufficient in limiting creep for the majority of indents, except those on very soft mastic. The effects of creep were present on the unloading curve for indents with a dwell time under 20 s. The Young’s modulus and hardness for samples with hydrated lime are higher than the samples without them. In the mastic phase, there is an increase of 31% in the Young’s modulus and 153% in the hardness due to the addition of hydrated lime. In the matrix phase, there is an increase of 6% in the Young’s modulus and 114% in the hardness due to the addition of hydrated lime. Future study on correlating the nanoscale testing with the macroscale testing presents exciting possibilities to improve current understanding of asphalt concrete. 

## Figures and Tables

**Table 1:** Mix details.

Materials (%)	Base (without Hydrated Lime)	With Hydrated Lime
10 mm aggregates	43.7	43.7
5 mm aggregates	11.4	11.4
Dust	28.5	28.1
Washed dust	11.4	10.4
Hydrated lime	0	1.5

**Table 2:** Properties of the mixes.

Properties	Values
Binder type	C320
Air voids	3.0–7.0%
VMA	≥16.0%
Stability	≥8.0 kN
Flow	2–4 mm
Compaction	75 blows
